# Simultaneous RNA Fluorescent In Situ Hybridization and Immunofluorescent Staining of Mouse Muscle Stem Cells on Fresh Frozen Skeletal Muscle Sections

**DOI:** 10.21769/BioProtoc.5435

**Published:** 2025-09-05

**Authors:** Vedant R. Lakkundi, Maria L. Perez, Kartik Soni, Albert E. Almada

**Affiliations:** 1 Department of Orthopaedic Surgery, Keck School of Medicine, University of Southern California (USC), Los Angeles, CA, USA; 2 Department of Stem Cell Biology and Regenerative Medicine (SCRM), Keck School of Medicine, University of Southern California (USC), Los Angeles, CA, USA; 3 Leonard David School of Gerontology, University of Southern California (USC), Los Angeles, CA, USA

**Keywords:** Immunofluorescence (IF), RNAscope, Fluorescent in situ hybridization (FISH), Muscle stem cells, Skeletal muscle, PAX7

## Abstract

Adult muscle stem cells (MuSCs) are the key cellular source for regenerating skeletal muscle in vertebrates. MuSCs are typically identified in skeletal muscle by the expression of the paired box protein 7 (PAX7) protein. Here, we developed a combined RNA fluorescent in situ hybridization (FISH) using RNAscope technology and an immunofluorescence (IF) protocol for the simultaneous detection of *Pax7* mRNA and PAX7 protein in individual MuSCs in vivo. Interestingly, we show that while most PAX7^+^ (protein) MuSCs express *Pax7* mRNA, there is a subset of *Pax7* ^+^ (mRNA) cells that do not express PAX7 protein. Altogether, we developed a combined FISH/IF protocol that allows for the co-detection of mRNA and protein in MuSCs in vivo, a strategy that can be applied to any target gene. The functional significance of the *Pax7*-expressing subset of cells lacking PAX7 protein prior to injury remains unknown.

Key features

• Extensive step-by-step details for an optimized protocol combining traditional immunofluorescence with RNA fluorescent in situ hybridization (FISH) using ACDBio’s RNAscope technology.

• Allows for the co-detection of protein and mRNA in muscle stem cells (MuSCs) in mouse skeletal muscle tissue in vivo in ~2 days.

• Validation of our protocol uncovers a subset of cells expressing *Pax7* mRNA but not PAX7 protein.

## Graphical overview


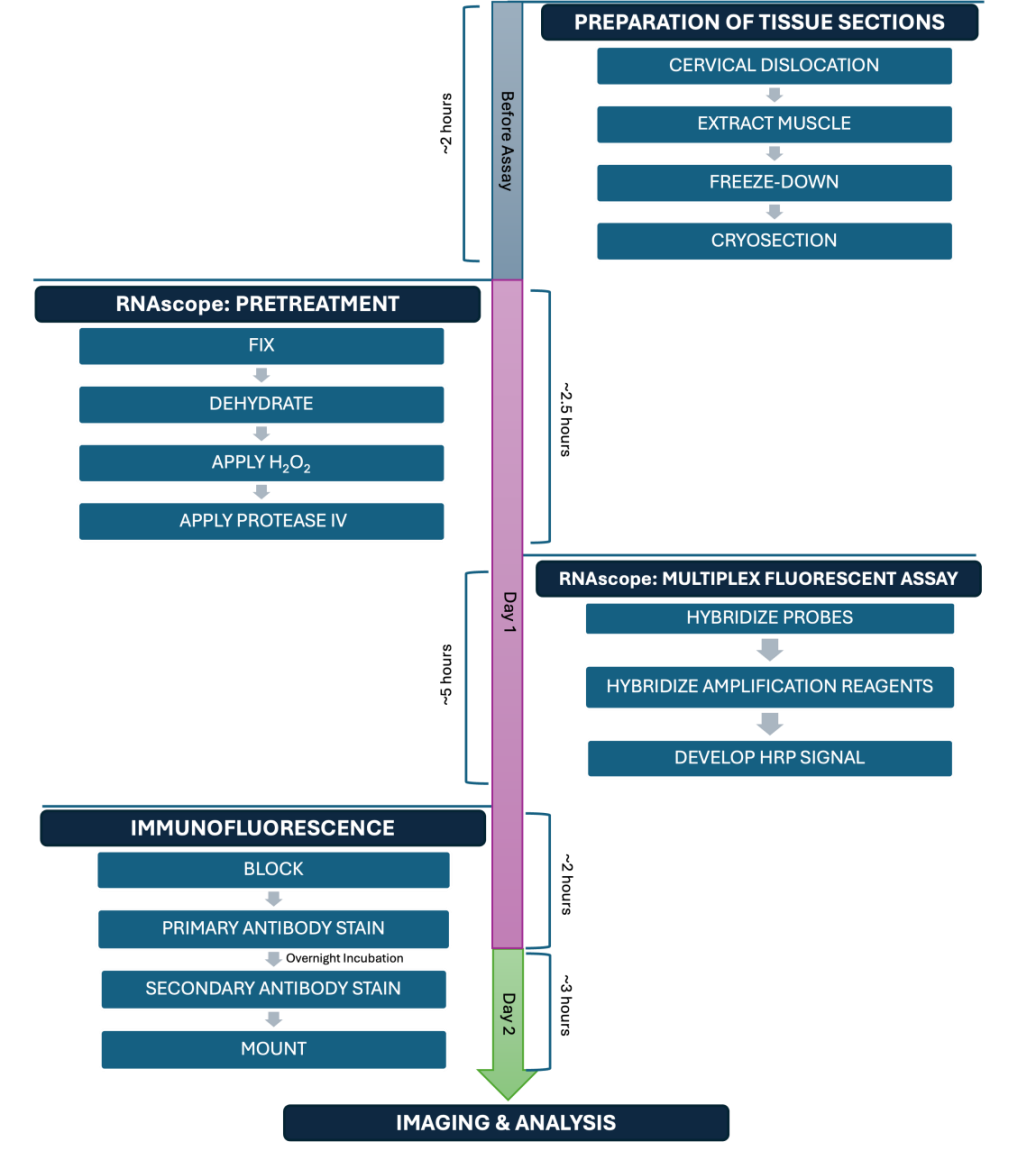


## Background

Muscle satellite cells (MuSCs) are a stem cell population responsible for regenerating skeletal muscle in vertebrate animals [1–6]. The paired box 7 (PAX7) protein is the canonical marker gene that is uniquely expressed by adult MuSCs in mammals [5]. The most common way of visualizing and quantifying PAX7-expressing MuSCs in skeletal muscle tissue before and after injury, in pathogenic states, or during aging in vivo is using immunofluorescence (IF) for PAX7 protein or with transgenic mice expressing a fluorescent protein using the Pax7 promoter (i.e., a fluorescent marker as a surrogate for PAX7 expression) [7].

A major limitation in studying MuSC fate decisions in mammalian tissue sections in vivo is the challenge in finding validated antibodies for performing a co-stain between PAX7 and your favorite protein of interest. While several good PAX7 antibodies exist, it can be difficult to find IF conditions that preserve PAX7 while also allowing for the detection of other antigens that indicate a change in differentiation, cell survival, cell proliferation, or other cell state changes. One potential way to address this need is to perform simultaneous detection of (1) PAX7 protein with IF and (2) RNA fluorescent in situ hybridization (FISH) of your favorite gene. Several companies have developed protocols for FISH, including ACDBio (RNAscope), Biosearch Technologies (Stellaris), and ViewRNA (Thermo Fisher Scientific), which vary in terms of probe chemistry, reagents used, and workflow.

Specifically, ACDBio commercialized a new technology called RNAscope [8], which is a proprietary type of RNA FISH that has gained popularity over the years. RNAscope is a technique that involves six major steps: 1) preparing your tissue sample; 2) pretreatment; 3) probe hybridization; 4) amplification steps; 5) labeling of sites where the probe is binding the target; and 6) detection of the signal. ACDBio established a streamlined RNAscope/IF protocol; however, there are several steps that need to be optimized for every RNA target and cell or tissue type. Indeed, a recent study developed an optimized RNAscope protocol for detecting protein and RNA simultaneously in single myofibers in culture ex vivo [9].

Here, we present a detailed RNAscope/IF protocol for performing simultaneous detection of mRNA and protein, using *Pax7* mRNA and PAX7 protein in MuSCs from uninjured skeletal muscle sections as an example. Overall, our protocol enables multiplexed analysis via the visualization of mRNA and protein targets within the same tissue section. Additionally, while performing our validation studies for this protocol, we discovered that while the majority of PAX7^+^ (protein) cells express *Pax7* mRNA, about 17% of cells that express *Pax7* mRNA have no detectable PAX7 protein. These data suggest that standard IF-based methods for detecting PAX7-positive cells alone likely miss a subset of *Pax7*-expressing cells that have not yet accumulated protein and whose functional significance remains unknown.

## Materials and reagents

**Biological materials**

1. Mice, C57BL/6J (*Mus musculus)* (The Jackson Laboratory, catalog number: 000664)

**Reagents**

1. Bovine serum albumin (BSA) (Sigma-Aldrich, catalog number: A9418-50G)

2. Normal goat serum (NGS) (VWR, catalog number: 102643-594)

3. Paraformaldehyde (PFA) (Electron Microscopy Sciences, catalog number: 15714-S)

4. M.O.M.^®^ blocking reagent (Mouse on Mouse) (Vector Laboratories, catalog number: MKB-2213-1)

5. Phosphate-buffered saline (PBS), 1× without calcium and magnesium (VWR, catalog number: 45000-446)

6. Hoechst 33342 solution, 20 mM (Life Technologies, catalog number: 62249)

7. Triton X-100 (VWR, catalog number: AAJ66624-AP)

8. Tween-20 (Fisher Scientific, catalog number: BP337-100)

9. Anti-PAX7 (Developmental Studies Hybridoma Bank, DSHB, catalog number: PAX7, RRID: AB_528428); see General note 1 for how we prepared the DSHB PAX7 antibody

10. Goat anti-mouse IgG1 cross-adsorbed secondary antibody, Alexa Fluor^TM^ 555 (Thermo Fisher Scientific, catalog number: A21127)

11. Reagent-grade 100% alcohol for histology (VWR, catalog number: 89370-084)

12. RNAscope^®^ Probe, Mm-Pax7, *Mus musculus* paired box gene 7 (Pax7), mRNA (Advanced Cell Diagnostics, catalog number: 314181)

**Caution:** This reagent contains formamide, which is carcinogenic.

13. RNAscope^®^ 3-plex positive control probe, Mm, RNAscope^®^ mouse positive control probe for RNAscope^®^ multiplex fluorescent assay, UBC, C3 channel (Advanced Cell Diagnostics, catalog number: 320881)

**Caution:** This reagent contains formamide, which is carcinogenic.

14. RNAscope^®^ 3-plex negative control probe, RNAscope^®^ negative control probe C1 channel dapB (of *Bacillus subtilis* strain) for RNAscope^®^ multiplex fluorescent assay (Advanced Cell Diagnostics, catalog number: 320871)

**Caution:** This reagent contains formamide, which is carcinogenic.

15. TSA Vivid^TM^ Fluorophore kit 650 (Bio-Techne, catalog number: 7527)

16. Nail polish, clear (Electron Microscopy Sciences, catalog number: 72180)

17. RNAscope^®^ Multiplex Fluorescent Reagent kit v2 (Advanced Cell Diagnostics, catalog number: 323100). From this kit, only the reagents listed in Table 1 were used.

**Table 1.** Key kit reagents for RNAscope

Sub-kit	Component
RNAscope^®^ H_2_O_2_ & Protease reagents (catalog number: 322281)	RNAscope^®^ hydrogen peroxide
	RNAscope^®^ protease IV
RNAscope^®^ Multiplex Fluorescent Detection Reagents V2 (catalog number: 323110)	RNAscope^®^ Multiplex FL V2 AMP 1*
	RNAscope^®^ Multiplex FL V2 AMP 2*
	RNAscope^®^ Multiplex FL V2 AMP 3
	RNAscope^®^ Multiplex FL V2 HRP-C1
	RNAscope^®^ Multiplex FL V2 HRP-C3
	RNAscope^®^ Multiplex FL V2 HRP blocker
	RNAscope^®^ Multiplex FL V2 DAPI
RNAscope^®^ TSA Buffer Pack (catalog number: 322809)	RNAscope^®^ Multiplex TSA buffer
RNAscope^®^ Wash Buffer Reagents (catalog number: 310091)	RNAscope^®^ wash buffer (50×)

*Caution: This reagent contains formamide, which is carcinogenic.

18. 2-Methylbutane (isopentane) (Sigma-Aldrich, catalog number: M32631-4L)

19. Tissue-Tek^®^ O.C.T. compound (VWR, catalog number: 25608-930)

20. Vectashield mounting medium (VWR, catalog number: 101098-042)

21. Dry ice

22. Liquid nitrogen

**Solutions**

1. 4% PFA in 1× PBS (see Recipes)

2. 1× wash buffer (see Recipes)

3. 50% histology-grade alcohol (see Recipes)

4. 70% histology-grade alcohol (see Recipes)

5. TSA Vivid Fluorophore 650 (see Recipes)

6. PBST (see Recipes)

7. 10% M.O.M. in PBST (see Recipes)

8. Blocking buffer (see Recipes)

9. Blocking buffer (with 1% M.O.M.) (see Recipes)

10. 1:2 Anti-PAX7 primary antibody in blocking buffer (see Recipes)

11. 1:500 Alexa Fluor 555 secondary antibody in blocking buffer (see Recipes)

12. 1 μg/mL Hoechst (see Recipes)

**Recipes**

**1. 4% PFA in 1× PBS**

**Table:** 

Reagent	Final concentration	Quantity or Volume
32% PFA	4%	12.5 mL
1× PBS	1×	87.5 mL
Total	N/A	100 mL

Store at 4 °C. Prepare fresh.

**2. 1× wash buffer**

**Table:** 

Reagent	Final concentration	Quantity or Volume
50× wash buffer	1×	60 mL
Double-distilled H_2_O	N/A	2.94 L
Total	N/A	3 L

Store at room temperature (RT) for up to 1 month.

**3. 50% histology-grade alcohol**

**Table:** 

Reagent	Final concentration	Quantity or Volume
Reagent-grade 100% alcohol for histology	50%	50 mL
1× PBS	1×	50 mL
Total	N/A	100 mL

Store at RT for up to 1 month.

**4. 70% histology-grade alcohol**

**Table:** 

Reagent	Final concentration	Quantity or Volume
Reagent-grade 100% alcohol for histology	70%	70 mL
1× PBS	1×	30 mL
Total	N/A	100 mL

Store at RT for up to 1 month.

**5. TSA Vivid Fluorophore 650**

**Table:** 

Reagent	Final concentration	Quantity or Volume
TSA Vivid Fluorophore	1:1,500	1 μL
TSA buffer	N/A	1.499 mL
Total	N/A	1.5 mL

Store at 4 °C. Prepare fresh.

**6. PBST**

**Table:** 

Reagent	Final concentration	Quantity or Volume
1× PBS	1×	299.7 mL
Tween-20	0.1%	300 μL
Total	N/A	300 mL

Mix with a stir bar. Store at RT for up to 1 month.

**7. 10% M.O.M. in PBST**

**Table:** 

Reagent	Final concentration	Quantity or Volume
PBST	N/A	1.8 mL
M.O.M.	10%	200 μL
Total	N/A	2 mL

Store at 4 °C. Prepare fresh.

**8. Blocking buffer**

**Table:** 

Reagent	Final concentration	Quantity or Volume
PBST	N/A	9.49 mL
BSA	3%	0.3 g
NGS	5%	500 μL
Triton X-100	0.3%	10 μL
Total	N/A	10 mL

Mix with a stir bar. Store at 4 °C. Prepare fresh.

**9. Blocking buffer (with 1% M.O.M.)**

**Table:** 

Reagent	Final concentration	Quantity or Volume
Blocking buffer	N/A	1.98 mL
M.O.M.	1%	20 μL
Total	N/A	2 mL

Store at 4 °C. Prepare fresh.

**10. 1:2 Anti-PAX7 primary antibody in blocking buffer**

**Table:** 

Reagent	Final concentration	Quantity or Volume
Anti-PAX7	1:2	750 μL
Blocking buffer (without M.O.M.)	N/A	750 μL
Total	N/A	1.5 mL

Store at 4 °C. Prepare fresh.

**11. 1:500 Alexa Fluor 555 secondary antibody in blocking buffer**

**Table:** 

Reagent	Final concentration	Quantity or Volume
Fluorophore-conjugated secondary antibody	1:500	3 μL
Blocking buffer (without M.O.M.)	N/A	1.497 mL
Total	N/A	1.5 mL

Shield from light. Store at 4 °C. Prepare fresh.

**12. 1 μg/mL Hoechst**

**Table:** 

Reagent	Final concentration	Quantity or Volume
PBST	N/A	1.9838 mL
1:100 Hoechst solution	1 μg/mL	16.2 μL
Total	N/A	2 mL

Hoechst stock used is 20 mM. Shield from light. Store at 4 °C. Prepare fresh.

**Laboratory supplies**

1. ImmEdge^TM^ hydrophobic barrier pen (VWR, catalog number: 101098-065)

2. KIMWIPES^TM^ delicate task wipers (VWR, catalog number: 21905-049)

3. Simport^TM^ Scientific EasyDip^TM^ slide staining jars (Fisher Scientific, catalog number: 22-038-489)

4. Simport^TM^ Scientific EasyDip^TM^ slide staining rack (Fisher Scientific, catalog number: 22-038-494)

5. Fisherbrand straight broad strong tip extra-long forceps (Fisher Scientific, catalog number: 16-100-107)

6. VWR^®^ Premium Superfrost^®^ Plus microscope slides (VWR, catalog number: 48311-703)

7. Disposable base mold or cryomold (Electron Microscopy Sciences, catalog number: 62352-15)

8. Secured hinge microscope slide boxes (Electron Microscopy Sciences, catalog number: 71470-W)

9. VWR^®^ precision tweezers (VWR, catalog number: 89259-984)

10. KIMAX^®^ griffin beakers (2 L), low form, double scale, borosilicate glass, Kimble Chase (VWR, catalog number: 89000-746)

## Equipment

1. VWR^®^ Signature^TM^ rocking platform shaker (VWR, catalog number: 12620-906)

2. ACD HybEZ^TM^ II hybridization system

a. HybEZ^TM^ II oven (Advanced Cell Diagnostics, catalog number: 321719)

b. HybEZ^TM^ humidity control tray with lid (Advanced Cell Diagnostics, catalog number: 310012)

c. EZ-Batch^TM^ slide holder with 20 slide capacity (Advanced Cell Diagnostics, catalog number: 310017)

d. EZ-Batch^TM^ wash tray (Advanced Cell Diagnostics, catalog number: 310019)

3. VWR^®^ bead bath (VWR, catalog number: 77553-322)

4. Leica CM 1860 UV cryostat

5. Zeiss LSM 800 Axio Imager.Z2 upright confocal microscope

## Software and datasets

1. Fiji (ImageJ) (Version 2.14.0, National Institute of Health, https://imagej.net/)

2. Adobe Illustrator (Version 29.2, https://www.adobe.com/home)

3. GraphPad Prism (Version 10.4.1, GraphPad Software, Inc., La Jolla, CA, Alternative: Excel Version 16.0, https://www.graphpad.com/)

4. ZEN (blue edition) (Version 3.4, https://www.zeiss.com/corporate/en/home.html)

## Procedure

Section A may be completed at any time prior to the subsequent steps. Sections B, C, and D1 must be completed on day 1; Section D2 on day 2; and Section E within one week of staining.

**A. Preparation of fresh-frozen tibialis anterior (TA) tissue sections**

1. Perform euthanasia according to your institution’s approved local animal ethics committee protocol and surgically harvest both TAs from the mouse as previously described [10].

2. Add O.C.T. gently to the cryomold edge to avoid bubbles. If bubbles form, remove them with a 200 μL pipette. Embed the TA into the O.C.T. using tweezers.

3. Place a 2,000 mL beaker with 300 mL of 2-Methylbutane into a larger ice bucket containing liquid nitrogen. Ensure that the level of liquid nitrogen is higher than that of 2-Methylbutane.

4. Cryo-preserve the TA by using extra-long forceps to carefully lower the cryomold in 2-Methylbutane cooled with liquid nitrogen for 45 s. Immediately transfer the cryomold to dry ice and store at -80 °C until needed for experimentation.

5. Collect 12 μm cryosections using a cryostat (we recommend Leica 1860 UV) from the entire length of the TAs, place on Superfrost Plus microscope slides, and then store slides in a secure slide box at -80 °C until required for studies (see General note 2).

**B. Preparation steps before starting the RNAscope-IF procedure**

**Figure 1.**
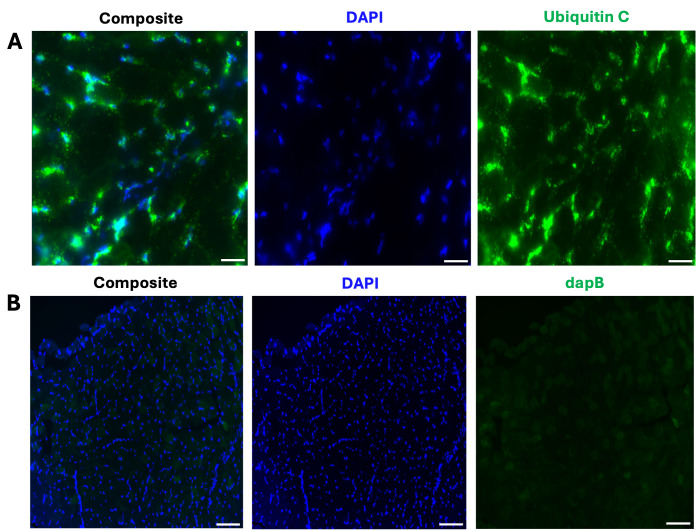
Representative images of the positive and negative control samples for the FISH assay. (A) Composite 40× image depicting the expression of ubiquitin C in a 12 μm transverse section from the tibialis anterior (TA). Scale bar: 50 μm. (B) Composite 20× image shows no *dapB* (bacterial gene) expression in a TA section. Scale bar: 100 μm. All representative images (A and B) were captured on the Zeiss LSM 800 Axio Imager.Z2 upright confocal microscope.

1. Chill 4% PFA in 1× PBS (see Recipe 1) at 4 °C for 1 h before beginning the procedure.

2. Prepare 2 L of 1× wash buffer (see Recipe 2). Ensure to warm the RNAscope 50× wash buffer to 40 °C using the bead bath for 10–20 min before diluting with double-distilled water.

3. Preset the HybEZ^TM^ II Oven to 40 °C.

4. Place tissue paper on the bottom of the HybEZ^TM^ humidity tray and fill it with 100 mL of ddH_2_O to dampen the paper. Remove any excess water.

5. Before steps C5–C9, bring H_2_O_2_, protease IV, amplification reagents (AMP 1–3), and horseradish peroxidase reagents (HRP-C1, HRP-C3, HRP blocker) to room temperature. H_2_O_2_ and protease IV are from the RNAscope H_2_O_2_ & Protease kit; AMP and HRP reagents are from the RNAscope Multiplex Fluorescent v2 kit.

6. Warm RNAscope probes at 40 °C in a bead bath for 10 min.

7. Critical controls to include in your experiment: ensure you have 1) a positive control slide where you apply the ubiquitin C control probe to validate the RNAscope reagents; 2) a negative control slide where you apply the dapB probe, which should not produce signal in mammalian tissues since it is a bacterial gene (Figure 1); and finally a 3) fluorophore-only negative control slide to ensure that no nonspecific binding of the Z probes occurs.

**C. Fluorescent in situ hybridization using RNAscope technology**

**Figure 2.**
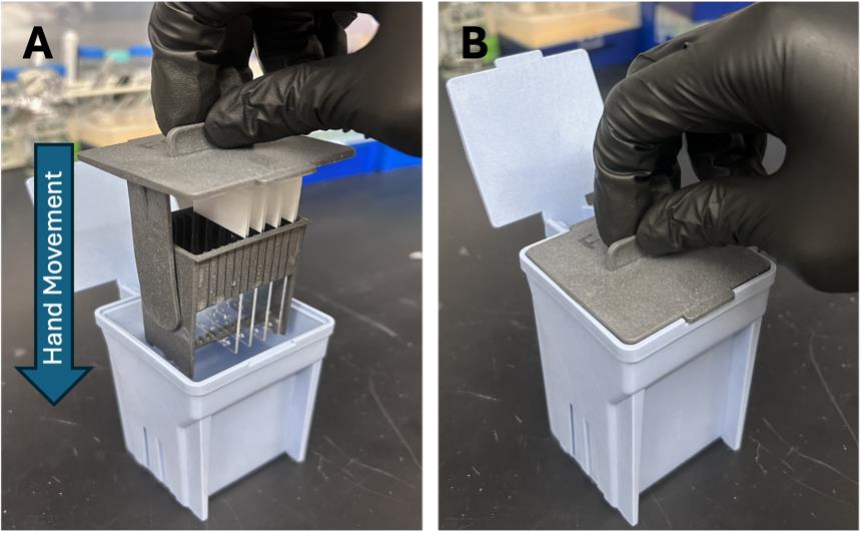
Slide staining system. (A) Insert the slide staining rack (with the slides secured) inside the slide staining jar. (B) Gently agitate via a series of up-and-down motions to remove excess 4% PFA from the slides.

1. Fixation of tissues on slides:

a. Remove slides from the -80 °C freezer and place inside a slide staining jar containing 100 mL of 4% PFA in 1× PBS. Chill at 4 °C for 1 h.

b. Fill two slide staining jars with 100 mL of 1× PBS each. Secure each slide inside a slide staining rack, which should fit inside the jar. In the first 1× PBS-filled jar, move the rack in an up-and-down motion to rinse the slides (containing the fixed sections) for 2 min (see Figure 2). Repeat again for the second jar.

2. Dehydration of tissues on slides:

a. Fill four slide staining jars with 100 mL of the following: 1) 50% histology-grade alcohol, 2) 70% histology-grade alcohol, 3) 100% histology-grade alcohol, and 4) a second time with 100% histology-grade alcohol.

b. Incubate the slides in 50% histology-grade alcohol for 5 min at RT.

c. Sequentially repeat dehydration steps (as in C2a, b) but instead use 70%, 100%, and then a second 100% wash, respectively.

**Figure 3.**
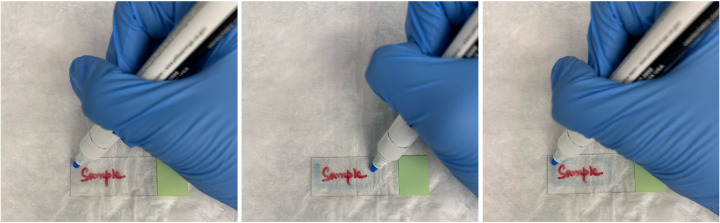
Visualization of how to draw the hydrophobic barrier around the histology slides

3. Remove the slides from the jar and let them air dry for 5 min on a Kimwipe at RT with the sections facing up.

4. Use the ImmEdge hydrophobic barrier pen to draw a barrier around all the sections being stained. It helps to re-trace the barrier three additional times to ensure it holds throughout the RNAscope/IF procedure. Air dry for 1 min at RT (see Troubleshooting, General notes 3 and 4, and Figure 3 to know barrier size and drawing instructions).

5. Pretreatment #1: Incubate slides with H_2_O_2_ to inactivate endogenous peroxidases, which ensures that the horseradish peroxidase (HRP) enzymatic reactions used for signal amplification are specific to RNAscope probes and not endogenous enzymes, improving the signal-to-noise ratio.

a. Load the slides onto the EZ-Batch^TM^ slide holder. Add five drops of H_2_O_2_ and ensure the sections for each slide are completely covered (see General note 3 to estimate the number of drops to add).

b. Incubate the slides for 10 min at RT in the humidity tray.

c. Perform a single wash step with 1× PBS (see General note 5 for details of the wash step).

6. Pretreatment #2: Incubate slides with protease IV to permeabilize cell membranes and release mRNA targets by degrading bound proteins. Protease IV works well for mouse muscle sections; however, protease standardization should be performed if another species or tissue type is used.

a. Add five drops of protease IV and ensure the sections for each slide are completely covered (see General note 3).

b. Incubate the slides for 15 min at RT in the humidity tray.

c. Perform a wash step with 1× PBS (see General note 5).

7. Probe hybridization between RNA probes and the specific target RNA:

a. Add five drops of appropriate probes and ensure the sections for each slide are completely covered (see General note 3).

b. Place the humidity tray inside the HybEZ^TM^ II oven that was preset to 40 °C and incubate for 2 h.

c. Remove the humidity tray. Repeat the wash step using 1× wash buffer (see General notes 5 and 6).

8. Probe amplification: Amplification at the site of probe/target binding to enhance the signal detection of the target RNA.

a. Add five drops of AMP 1 and ensure the sections for each slide are completely covered (see General note 3).

b. Place the humidity tray inside the HybEZ^TM^ II oven preset to 40 °C and incubate for 30 min.

c. Remove the humidity tray. Repeat the wash step using 1× wash buffer (see General notes 5 and 6).

d. Repeat steps C8a–c for AMP 2 (30 min at 40 °C) and finally AMP 3 (15 min at 40 °C).

9. Use horseradish peroxidase (HRP) to catalyze the attachment of fluorescent TSA Vivid Fluorophores at the target site, which results in greater signal intensity.

a. Add five drops of HRP-C1 and HRP-C3 and ensure the sections for each slide are completely covered (see General notes 3 and 7).

b. Place the humidity tray inside the HybEZ^TM^ II oven that was preset to 40 °C and incubate for 15 min.

c. Remove the humidity tray. Repeat the wash step using 1× wash buffer (see General notes 5 and 6).

d. Add five drops of TSA Vivid Fluorophore 650 (1:1,500, see Recipe 5) and ensure the sections for each slide are completely covered (see General note 3).

e. Place the humidity tray inside the HybEZ^TM^ II oven that was preset to 40 °C and incubate for 30 min.

f. Remove the humidity tray. Repeat the wash step using a 1× wash buffer (see General notes 5 and 6).

g. Add five drops of HRP-blocker and ensure the sections for each slide are completely covered (see General note 3).

h. Place the humidity tray inside the HybEZ^TM^ II oven preset to 40 °C and incubate for 15 min.

i. Remove the humidity tray. Repeat the wash step using 1× wash buffer (see General notes 5 and 6).

For positive and negative control slides that only require RNAscope, complete steps in General note 8 before proceeding to IF.

**D. Immunofluorescence (IF)**

1. Day 1

a. Transfer the slides to a slide box and wash with PBST at RT for 5 min 4 times (see General note 9).

b. Apply 150 μL of 10% M.O.M. in PBST (see Recipe 7) to cover the sections for each slide. Incubate for 30 min at RT (see General note 3).

c. Apply 150 μL of blocking buffer with M.O.M. (see Recipe 9) to cover the sections for each slide. Incubate for 1.5 h at RT (see Troubleshooting and General note 3).

d. Apply 150 μL of primary PAX7 antibody (1:2) in blocking buffer (see Recipe 10) to cover the sections for each slide. Incubate at 4 °C for 12–16 h (overnight) (see General note 3).

2. Day 2

a. Wash with PBST at RT for 15 min 4 times (see General note 9).

b. Apply 150 μL of Alexa Fluor^TM^ 555 secondary antibody (1:500) in blocking buffer (see Recipe 11) to cover the sections for each slide. Incubate at RT for 45 min (see General note 3).

c. Wash with PBST at RT for 15 min 4 times. During the third wash, counterstain with the Hoechst solution (see General notes 9 and 10 and Recipe 12).

d. Mount each slide with 25 μL of Vectashield mounting medium.

e. Carefully apply coverslips over the sections for each slide. Seal the coverslips on all sides three times using nail polish. Dry at RT for 5 min (see General note 11).

f. Store slides at 4 °C for up to 1 week and shield from light.

See “Troubleshooting” if issues arise relating to high background or low signal intensity when imaging.

**E. Image acquisition**

1. Acquire images using a Zeiss LSM 800 Axio Imager.Z2 upright confocal microscope. To quantify the number of double-positive cells or to take representative images, set the magnification to 20× or 63×, respectively.

2. Ensure the master gain, laser power, and acquisition parameters (averaging, bits per pixel, direction, scan speed, frame size, etc.) are adjusted appropriately, making sure no signal is observed in the control slides, and remain constant for all images.

3. Acquire Z-Stacks every 1.5 μm with 8–10 stacks per image.

4. Perform orthogonal projection for each image captured using the processing feature of ZEN software.

## Data analysis

To validate this protocol, three uninjured mice (N = 3) were used in this experiment. For each replicate, three depths from the mid-belly of the tibialis anterior (TA) were co-stained for PAX7 protein, *Pax7* mRNA, and Hoechst (nuclei). The use of multiple biological replicates and evaluating multiple depths from a single TA allowed us to control for biological and technical variability within a sample.

For each depth, 63× representative images and a 20× tile scan were taken on the LSM 800 confocal microscope. Due to the presence of *Pax7* mRNA and PAX7 protein on different Z-planes in a singular depth, the maximum projection was captured for each image. To generate images for quantification, a 4×4 tile scan using a 20× objective was imaged at the center of each muscle section.

**Figure 4.**
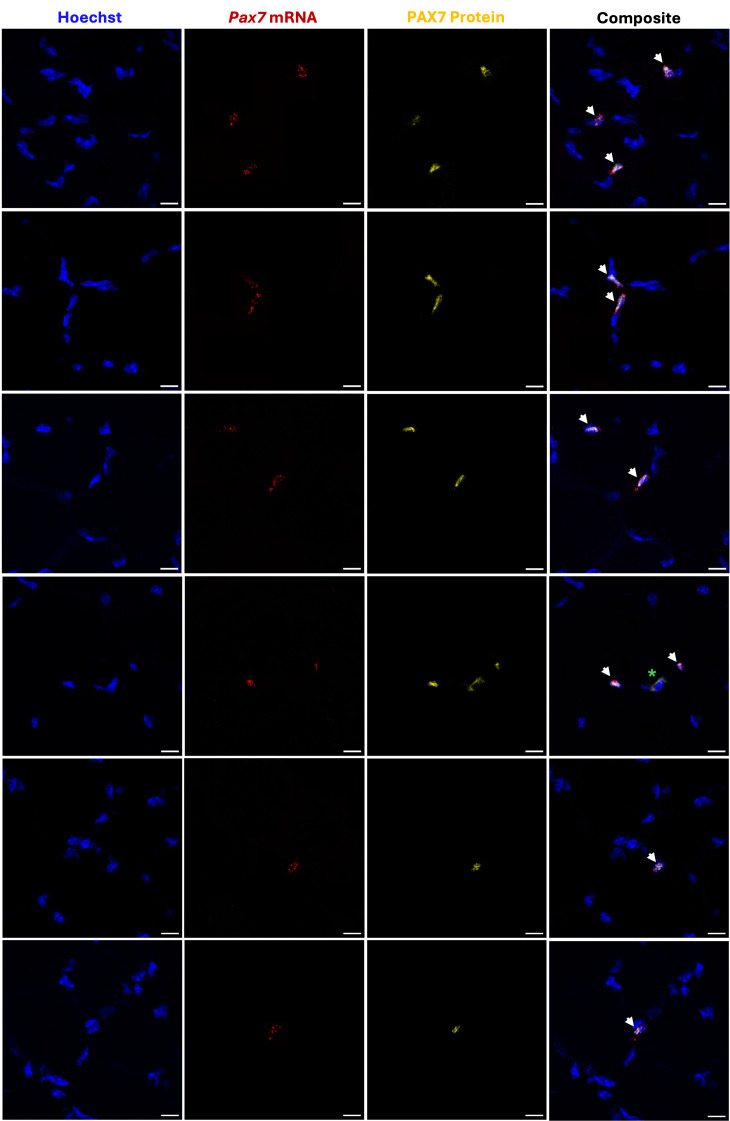
Representative images of adult muscle stem cells (MuSCs) double positive for *Pax7* mRNA and PAX7 protein. Several images were taken at 63× magnification on a Zeiss LSM 800 Axio Imager.Z2 upright confocal microscope. Each zoomed 12 μm transverse section depicts 1–3 double-positive cells. *Pax7* mRNA is indicated by the presence of numerous punctate dots. *Pax7* mRNA and PAX7 protein co-localize with Hoechst. Green asterisk denotes rare nonspecific staining that does not constitute positive signal, as it does not overlap with Hoechst. Scale bar: 10 μm.

**Figure 5.**
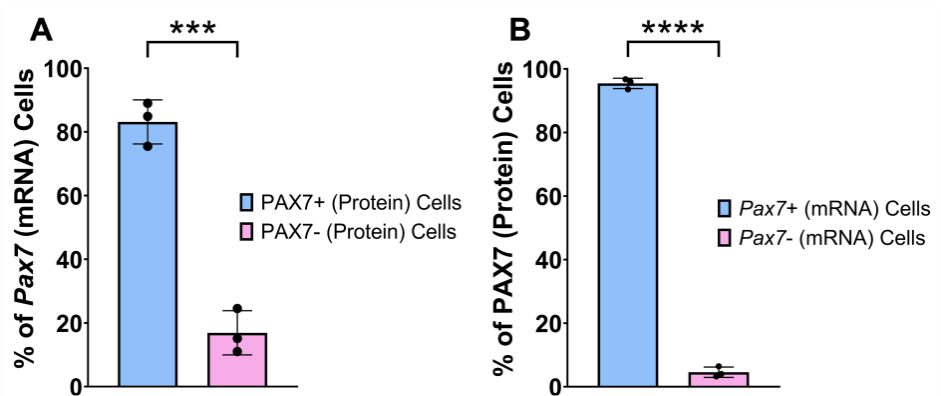
Quantification of PAX7 protein and *Pax7* mRNA expression in adult muscle stem cells (MuSCs) from uninjured skeletal muscle. (A) Percentage of cells expressing *Pax7* mRNA that also express PAX7 protein (PAX7+cells) or do not express PAX7 protein (PAX7- cells) in the 4×4 20× tile scan. (B) Percentage of cells expressing PAX7 protein that also express *Pax7* mRNA (*Pax7*+ cells) or do not express *Pax7* mRNA (*Pax7*- cells) in the 4×4 20× tile scan. The notation for protein and mRNA is PAX7 and *Pax7*, respectively. Data are presented as mean ± SD. Mean populations were compared using a Student’s unpaired, two-tailed t-test. ****p < 0.0001, ***p < 0.001.

The cell counter program in Fiji software was used for quantification. Each tile scan was imported into Fiji, and different colors were assigned to each channel: Hoechst (channel 1: blue), *Pax7* mRNA (channel 2: red), and PAX7 protein (channel 3: yellow). Adjustments to brightness and contrast were applied equally across each tile scan, and a grid was applied to aid counting. The number of double-positive cells was manually counted first, followed by *Pax7* ^+^ (mRNA) cells, and finally PAX7^+^ (protein) cells. While both the *Pax7* mRNA and PAX7 protein were present in the Hoechst-stained nuclei, the mRNA appeared as numerous punctate dots, while the protein typically overlapped the entire Hoechst stain in each MuSC (Figure 4). Four calculations were completed for each depth: (1) percentage of total *Pax7* (mRNA) cells that express PAX7 (protein); (2) percentage of total *Pax7* (mRNA) cells that do not express PAX7 (protein); (3) percentage of total PAX7 (protein) cells that express *Pax7* (mRNA); and (4) percentage of total PAX7 (protein) cells that do not express *Pax7* (mRNA). The average was taken across the three depths for each replicate for each specific calculation, and resulting data was plotted in GraphPad Prism (Figure 5).

## Validation of protocol

From our validation analysis, we found that 83.12% of *Pax7^+^* (mRNA) cells also express PAX7 (protein), consistent with the coordinate expression of *Pax7* mRNA and PAX7 protein. However, there also appears to be 16.88% of *Pax7^+^* (mRNA) cells that do not express the protein, suggesting that traditional IF methods miss out on a significant number of MuSCs that have not yet accumulated the PAX7 protein. On the other hand, when we first look at PAX7^+^ (protein) cells, 95.44% of them also express *Pax7* mRNA, as expected, but 4.56% of PAX7^+^ cells (protein) do not express the mRNA, suggesting that this small subset of cells may already be transcriptionally suppressing *Pax7* expression to promote activation [11]. Future studies should focus on understanding the identity and functionality of the *Pax7*-positive cells that do not yet express protein.

## General notes and troubleshooting

**General notes**

1. You can obtain a detailed protocol for harvesting the PAX7 antibody from DSHB hybridoma cells from the manufacturer (https://dshb.biology.uiowa.edu/PAX7).

2. The investigator will need prior training using the cryostat in their institution if they do not have a dedicated technician who prepares the cryosections.

3. For each slide, we drew a 0.75-inch × 0.75-inch hydrophobic barrier of size around 3 muscle cryosections, requiring 5 drops or 150 μL of the reagent to cover the muscle sections completely. We recommend measuring the length and width of the barrier drawn and using the calculated area to estimate the number of drops or microliters to add. Drawing the smallest possible hydrophobic barrier around the sections preserves reagents and reduces costs.

4. We recommend referring to Figure 3 when drawing the hydrophobic barrier.

5. RNAscope requires several wash steps with either 1× PBS or 1× wash buffer. The following instructions comprise a wash step:

a. Pour 200 mL of 1× PBS or 1× wash buffer into the EZ-batch wash tray.

b. Place the slide holder inside the wash tray. Set the rocker to 25 speed tilt and rinse for 2 min at RT. Replace with fresh 1× PBS or 1× wash buffer and repeat the 2-min rinse at RT.

c. Pour out the 1× PBS or 1× wash buffer, returning the slide holder to the humidity tray for next use.

For simplicity, these steps will be referenced throughout the protocol as “Perform a wash step” with either 1× PBS or 1× wash buffer specified.

6. Although the wash buffer of some FISH assays contains formamide, this chemical is not used in RNAscope according to the safety data sheet published by ACDBio.

7. HRP-C1 reagent should be applied to all slides that contain probes in Channel 1. HRP-C3 reagent should be applied to all slides that contain probes in Channel 3. Probes can be assigned to either Channel 1, 2, or 3 when purchased from Advanced Cell Diagnostics (ACDBio). For our study, the PAX7 probe and dapB probe (negative control) were Channel 1 probes, while the ubiquitin C probe (positive control) was a Channel 3 probe. Different HRP channels (C1, C2, or C3) are used to ensure that each HRP enzyme is restricted to its respective C1, C2, or C3 probe, ensuring the accurate binding of fluorophores.

8. If only RNAscope is required for positive and negative control slides, the following steps should be followed instead of proceeding with immunofluorescence:

a. Apply five drops of DAPI (provided with the Multiplex Fluorescent Detection kit) to cover the sections for each slide. Incubate for 30 s at RT.

b. Mount each slide with 25 μL of Vectashield mounting medium.

c. Carefully apply coverslips over the sections for each slide. Seal the coverslips on all sides three times using nail polish. Dry at RT for 5 min.

d. Store slides at 4 °C for up to 1 week and shield from light.

9. The wash step for IF is different than that for RNAscope. For IF, apply 150 μL (or enough to cover the muscle sections) of PBST to the sections. After the specified wash time, gently tap the side of the slides on a Kimwipe to remove the reagent.

10. DAPI was used for RNAscope-only slides (ubiquitin C positive and dapB negative control) according to ACDBio’s instructions and the reagents provided in the Multiplex Fluorescent Detection Reagents V2 kit. Our conventional immunofluorescence protocol uses Hoechst for counterstaining, explaining its use in this part of the protocol.

11. To prevent bubble formation, ensure the mounting media is free of bubbles. Hold the coverslip at a 45° angle, with one edge touching the media, and gradually lower it to allow the media to spread evenly. If bubbles form, use a clean pipette tip to gently guide them to the edge.

**Troubleshooting**

**Table:** 

Problem	Solutions
Reagents leaking through the hydrophobic barrier	• Ensure the ImmEdge pen has a wet tip when drawing a barrier around the sections of interest. • (Optional) Reapply the hydrophobic barrier between RNAscope and IF or when the barrier breaks.
Visualizing weak RNAscope and/or IF signal when imaging	• Image within 2 days of completing day 2 of the IF section of the protocol. • Ensure probes and antibodies are not expired. • Store antibody mixes on ice and shielded from light before applying to sections.
Observing high background signal when visualizing PAX7 protein in its specific channel	• Ensure there are no morphological or artificial defects in muscle sections when freezing down. • Prevent sections from drying out primarily between the dehydration and hydrophobic barrier step.
